# Evolution of Excited States in Bismuth Vanadate: Trapping and Kinetic Pathways

**DOI:** 10.1021/acs.jpclett.6c00396

**Published:** 2026-04-10

**Authors:** Tobias Möslinger, Julia Wiktor

**Affiliations:** 11248 Chalmers University of Technology, Department of Physics, 41296 Gothenburg, Sweden

## Abstract

Photoelectrocatalytic water splitting using bismuth vanadate (BiVO_4_) is a promising approach for sustainable hydrogen production, but its efficiency is limited by charge carrier dynamics. Though charge trapping in the form of polarons is well-studied, the behavior of self-trapped excitons (STEs), particularly whether they remain stable or dissociate under operating conditions, remains far less understood. Using hybrid density functional theory with the nudged elastic band method, we quantify activation barriers for STE hopping, dissociation and transformation in BiVO_4_, revealing distinct behaviors and kinetic time scales for two STE types: a separated, more mobile state and a compact, more stable one with higher barriers. Additionally, we study an alternative charge trapping mechanism via O–O dimers, providing an alternative multipolaron binding pathway with distinct kinetics. These findings provide fundamental insights into the kinetic stability and mobility of trapped charges in BiVO_4_, aiding the interpretation of charge trapping dynamics under operating conditions.

## Graphical abstract

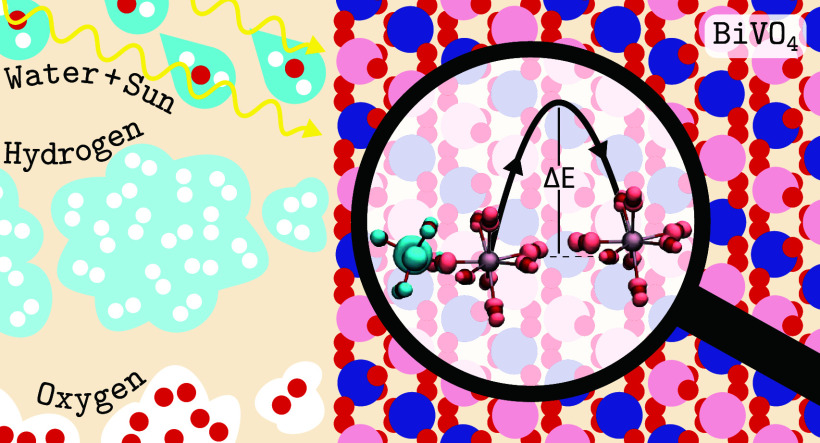

Bismuth vanadate (BiVO_4_) has emerged as one of the most promising photoanode materials for photoelectrocatalytic water splitting, thanks to its favorable band gap of approximately 2.4 eV, which aligns well with the requirements for visible light absorption and water oxidation.^1–3^ Despite its potential, the practical efficiency of BiVO_4_ is hindered by poor charge transport and rapid recombination of photogenerated electron–hole pairs.^4–7^ These limitations are closely tied to the behavior of excess charge carriers in the material, which is still an active area of research.^8–10^

In oxide semiconductors, excess charge carriers tend to localize, leading to lattice distortions and the formation of polarons—quasiparticles that occupy energy levels within the band gap and can interact with other carriers and defects.^11–13^ Charge localization has been experimentally observed in BiVO_4_,^14^ where electrons and holes exhibit distinct dynamics: electrons rapidly collapse into localized small polarons, while holes initially remain more delocalized before being captured over longer time scales.^15,16^ Polaron formation impedes charge transport by localizing individual carriers, and can promote further charge trapping via electron–hole binding into self-trapped excitons (STEs).^17–19^ Enhancing photoinduced charge separation is therefore a key goal for improving photoanode performance.^20^

Recent studies have highlighted the formation of STEs in BiVO_4_ as an additional pathway for charge trapping.^12,15,21,22^ We have recently computationally identified two distinct types of STEs, both with similar formation energies, suggesting a complex interplay between localized electron and hole polarons and lattice distortions.^23^ Alongside STEs, the formation of O–O hole dimers has been proposed in ref 24 as another mechanism for charge trapping, potentially explaining the delayed dynamics observed in spectroscopic experiments. Such multihole binding may introduce slower kinetics than STE formation and therefore provides an important alternative trapping pathway. However, the behavior of these trapped states, including their stability, mobility and propensity for dissociation, remains poorly understood, leaving critical gaps in our understanding of charge transport limitations in BiVO_4_. In particular, without quantitative activation barriers, it is not possible to assess whether STEs are expected to remain intact, migrate as bound pairs, dissociate into separate polarons, or transform between configurations on experimentally relevant time scales.

To address these open questions, this work combines density functional theory (DFT) with hybrid functionals to calculate the structures of STEs and their energy barriers in different processes in BiVO_4_. Nudged elastic band (NEB) calculations are used to investigate their mobility, stability and the energy barriers associated with pairwise hopping, electron–hole separation, or transformation between STE types. By elucidating these processes, our findings provide a barrier-based mechanistic picture of STE stability and mobility under operating conditions, with implications for enhancing the overall efficiency of water splitting.^25,26^

The calculations presented here are performed within the CP2K code.^27^ We apply the PBE0­(α) hybrid functional with a fraction of α = 14% of exact Hartree–Fock exchange to relax the initial and final points of the process and carry out NEB calculations.^28^ The α value of 14% is derived in ref 23 from Koopmans’ condition and applied here due to its relevance for localized states. For more computational details and convergence tests, see the Supporting Information (SI).

The energy barriers for different analyzed processes are presented in relation to the configurational coordinate Q of the corresponding structure, calculated as

1 $$Q_{i}=\sqrt{\underset{j}{\sum }m_{j}{\left|r_{i,j}-r_{0,j}\right|}^{2}}$$

Here, *i* indexes the NEB image, while *m*_*j*_ and *r*_*i, j*_ are the mass and position of atom *j*.^29^ Schematic representations of the initial and final structures with their charge isosurfaces can be found in the SI as insets in the graphs.

To analyze the time delays related to different barriers, we use the Arrhenius equation for the rate *k* of a thermally activated process

2 $$k=\kappa \nu \Gamma \;\exp\left(-\frac{E}{k_{B}T}\right)$$

with the activation energy *E*, the Boltzmann constant *k*_B_, the temperature *T* (room temperature was assumed) and the attempt frequency ν.^30^ The electronic transmission coefficient κ is defined as

3 $$\kappa =\frac{2P}{1+P}$$

We here assume the adiabatic regime, in which *P* → 1, and thus κ = 1, but keep nonadiabatic effects in mind when discussing the results. Furthermore, the nuclear tunneling factor Γ should only be important for low temperatures or light elements and can therefore also be set to 1.^29,31^ The attempt frequency can be obtained from the second derivative of the energy with respect to the configurational coordinate at the polaron ground state *Q*_0_:

4 $$\nu =\sqrt{\frac{\partial^{2}E\left(Q\right)}{\partial Q^{2}}}{|}_{Q=Q_{0}}$$

The time scale τ is found with the inverse of the rate

5 $$\tau =\frac{1}{k}$$

We note that the resulting time scales are intended as order-of-magnitude estimates to compare competing mechanisms, rather than absolute predictions. The detailed values are presented in the SI in Table S3.

The investigations in this study focus on the two types of STEs found previously^23^ and later also consider the O–O dimer configuration.^24^ The shape of both STE types is shown in Figure 1. STE1 exhibits a more separated localization, where the electron is positioned around a V atom in the *d*_*z* ^2^_-orbital shape, while the hole is arranged around the closest neighboring Bi atom. STE2 on the other hand is more compact, with the electron around the V atom in the *d*_*xy*_-orbital shape, but the hole localized now around an O atom of the same VO_4_ unit. Formation energies previously found by us^23^ within VASP are – 0.88 eV for the STE1 and – 0.85 eV for the STE2. Calculations performed within CP2K show similar formation energies for both types of STEs. However, the order is now changed, with STE2 being slightly more stable at – 0.86 eV than STE1 at – 0.82 eV. Nevertheless, these energy differences observed are comparable to the typical uncertainties associated with these computational methods. The relative ordering is thus sensitive to the computational setup (here, VASP vs CP2K) and, as shown in ref 23, also to the fraction of exact exchange α. We therefore treat the small differences in stability with caution when discussing transformation kinetics. To test the dependence of energy barriers on α, we perform additional calculations with α = 22% on the STE1 hopping barrier and the transformation between STE2 and STE1 (see the SI).

**Figure 1:**
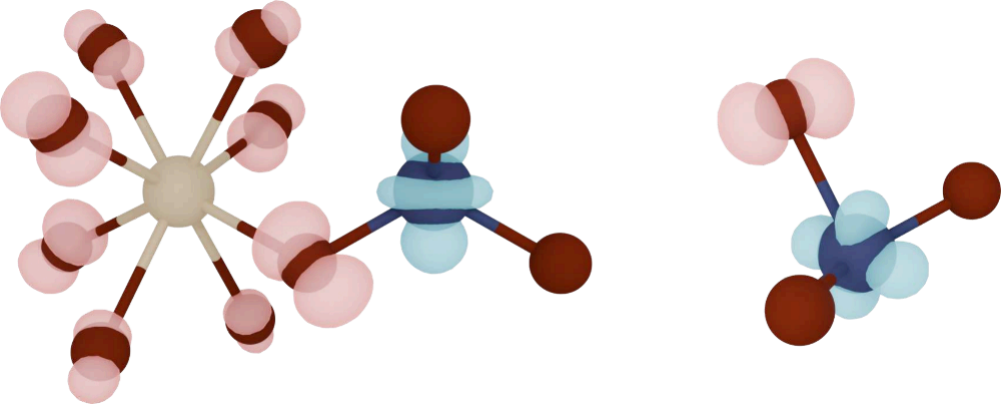
Drawing of STE1 (left) and STE2 (right) found in BiVO_4_ with their charge densities. Bi, V and O atoms are colored in beige, dark blue and dark red, respectively, while the charge isosurfaces for the electron and hole are shown in turquoise and pink.

To understand how STEs in BiVO_4_ evolve after formation, we compute activation barriers for (i) hopping of the full STE (electron and hole) between neighboring sites, (ii) dissociation into separate electron and hole polarons and (iii) transformations between STE configurations. We use NEB calculations to obtain energy barriers for these pathways and systematically explore relevant structural rearrangements to map the associated energetics. Sketches of the considered paths are provided as insets in Figure 2. The individual processes are discussed in more detail in the following.

**Figure 2:**
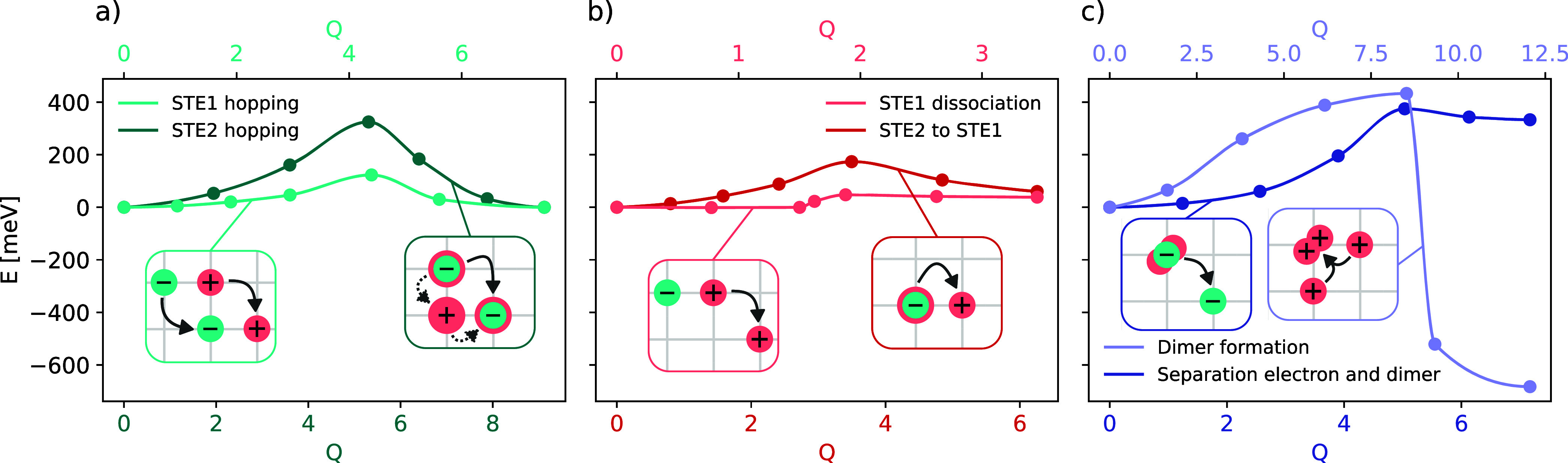
Plots of the activation barriers for investigated paths: (a) hopping, (b) dissociation and transformation, (c) dimer processes. The insets show schemes of how electron (turquoise) and hole (pink) move in the material.

S*TE Kinetic Pathways*. In the event that the STEs are mobile and transported as a whole, their mobility would be determined by the energy required to induce their hopping to another position within the material. Therefore, we investigate the barrier that needs to be overcome for both the electron and hole to migrate simultaneously (process i). The initial and final positions for the STEs are chosen so that hopping occurs along the shortest possible path. For the STE1, the electron and hole hop between neighboring V and Bi sites, respectively, while remaining localized around separate atoms (see Figure S2a). For the STE2, the electron and hole move together from the same starting atom to the nearest-neighbor V atom (see Figure S2b). We find an energy barrier of approximately 123 meV for the hopping of STE1, while for the STE2 it is significantly higher at 322 meV. A comparison of these energies with other analyzed processes is provided in Table 1, while the full paths are shown in Figure 2. From the energy barriers, using eqs 2 and 5, we estimate the time delay for the hopping of STE1 in the picosecond range. Due to the much higher barrier for the hopping of STE2 and the exponential dependence, the delay amounts to a much longer time in the nanosecond range for this process. We note that our analysis assumes an adiabatic regime when estimating STE hopping rates. For strongly localized carriers, nonadiabatic effects can reduce hopping rates if the electronic coupling between sites is small compared to the lattice reorganization energy.^32,33^ Such effects are expected to be more relevant for the compact and high-barrier STE2, whereas the more extended lattice distortion and lower hopping barrier of STE1 make the adiabatic approximation more reasonable.^34^ Although nonadiabatic corrections may affect absolute hopping rates, the qualitative trend of STE1 being significantly more mobile than STE2 is unlikely to change.

**Table 1:** Energy Barriers for Different Hopping, Dissociation or Transformation Mechanisms^a^

Type of process	*E* [meV]
STE1 hopping	123
STE2 hopping	322
STE1 formation	9
STE1 dissociation	48
STE2 to STE1	174
STE1 to STE2	113
	
Dimer formation	389
Dimer separation	1071
Dimer - electron trapping	42
Dimer - electron separation	377

a All calculations were done with CP2K.

An explanation for the significant difference between the two states can be found by examining the entire simulation path step-by-step. In the case of STE1, the transition state is achieved by temporarily delocalizing the hole, which then localizes around a new atom. Only then, the electron follows to hop to the new site as well, leading to two separate processes. Because the hole is weakly bound and can easily migrate, the required energy of the entire process is determined mainly by the electron mobility. Conversely, the hopping path for the STE2 proves more complex. Observing the transition state reveals that the electron and hole cannot jump to a new position together when localized around the same V atom. Therefore, the transition state requires the hole to break away from the electron and localize around a nearby Bi atom. The electron can then jump to the final position once the hole has moved away, maintaining the structure characteristic for the STE2 state. Finally, the hole rejoins the electron at the new atom, completing the migration of STE2 to the new location. This process explains the much higher activation energy, as the separation and rebinding of the hole and electron nearly constitute two transformations from STE2 to STE1 and back, which will be analyzed in detail in the subsequent sections.

Because of the relatively high energy barriers of the hopping processes and the previously reported low binding energies of the STEs,^23^ we also investigate their dissociation (process ii). First, we focus on the dissociation of STE1, in which the hole is moved away from the electron polaron from the first to second neighbor Bi (see Figure S3a). The energy barrier for this process is 48 meV (Figure 2b). This low barrier indicates that the hole can detach from the electron at modest energetic cost, suggesting that STE1 is only weakly kinetically stabilized against dissociation. The corresponding Arrhenius estimate yields a dissociation time scale of around a picosecond. In the reverse direction, the barrier for STE1 formation from separated electron and hole polarons is only 9 meV, implying an even faster formation time scale in the subpicosecond range. For STE2, attempting to separate the hole from the electron directly leads to relaxation into an STE1-like configuration. As any way of moving the hole away from the electron in this state immediately leads to the configuration changing into the arrangement of STE1, the transformations between the two types are studied instead (process iii).

Initially, we study the transformation from STE2 to STE1. The relaxed path shows an increase in the system’s total energy until the barrier is reached, allowing the hole to detach from the electron. Following that, the hole settles around the closest possible Bi atom to the V. The electron remains on the same V atom throughout the process, but changes the shape of its orbital from *d*_*xy*_ to *d*_*z* ^2^_. However, charge delocalization, as observed during the hopping of the STE1, cannot be seen in this case (see Figure S3b). The activation barrier for this process is 174 meV (Figure 2b), consistent with the relative ordering of STE formation energies obtained in CP2K. Pathways involving electron relocation are expected to be higher in energy and are therefore not explored further. The reverse process, the transition from the STE1 state to the STE2 state, is found to have an activation barrier of 113 meV. We note that the relative stability of the two STE configurations depends on the computational setup: in our previous work using VASP with α = 14%, STE1 was favored by 30 meV, whereas the present CP2K calculations favor STE2 by 40 meV.^23^ This sensitivity suggests that the relative transformation barriers may change accordingly. The resulting barriers correspond to estimated time delays in the range of picoseconds for the transformation from STE2 to STE1 and for the reverse process.

To place our results in context, we now summarize relevant experimental observations on charge trapping dynamics in BiVO_4_. Direct observations of STE migration are not available; however, a study by Zhang et al.^15^ investigated the dynamics of excited-state polaronic trapping using transient absorption (TA) and time-resolved terahertz (TR-THz) spectroscopies. The TA spectra in the 0.75 to 1 eV range revealed the evolution of free holes into localized states over time, which has been associated with the emergence of long-lived trapped states, potentially including STE formation. They concluded that electrons in the conduction band collapse into severely localized small electron polarons on a subpicosecond time scale, while holes in the valence band remain delocalized initially but are subsequently captured by electron polarons to form STEs on longer time scales. Other TA spectroscopy measurements presented by Ravensbergen et al.^16^ show different absorption times. They observed trapping of a small fraction of holes after 0.12 ps, while the majority of holes is trapped within 5 ps. Furthermore, they report electrons undergoing relaxation with a time constant of 40 ps before deeper trapping on the 2.5 ns time scale, while trap-limited recombination extends from 10 ns to 10 μs.

Comparing these observations with our estimated time scales, we find very good agreement between the experimentally reported fast hole trapping and the calculated formation times of STE1 (subpicosecond) and STE2 (picoseconds). We here assume that STE2 formation occurs via STE1, such that the effective formation time scale is approximated by the STE1-to-STE2 transformation time. The transformation times between the two STE configurations (in the range of picoseconds) are comparable to the electron relaxation time reported by Ravensbergen et al.^16^ While STE1 hopping (picoseconds) falls in the same range, in contrast, STE2 hopping occurs on much longer time scales (nanoseconds), comparable to trap-limited recombination.

At the same time, the observation of long-lived trapped charges persisting into the nanosecond to microsecond regime^16^ suggests that, beyond the STE-related processes considered here, additional trapping configurations with slower kinetics may become relevant. To assess such possibilities, we therefore explore alternative charge-binding mechanisms below.

*O–O Hole Dimers as Alternative Binding Pathway*. In addition to the binding between one electron and one hole within STEs, we investigate two alternative mechanisms: (iv) the formation of multihole bound states in the form of hole dimers from two localized holes in close proximity and (v) electron trapping at the dimer, and the corresponding detrapping (electron release) to a neighboring site. Both of these processes may occur in experiments and exhibit slower kinetics due to larger structural rearrangements. The formation of this O–O hole dimer in BiVO_4_ was previously investigated in ref 24. The structure is shown in Figure 3. We find a formation energy *E*_f_ of about −1.0 eV (−0.5 eV per hole) for the O–O dimer in the singlet state. Here, *E*_f_ = *E*_dimer_ – *E*_pristine_ + 2·ϵ_VBM_ + *E*_corr_, using the total energy of the cell with a dimer *E*_dimer_ and in the pristine state *E*_pristine_, as well as the energy of the valence band maximum ϵ_VBM_ and a finite-size energy correction *E*_corr_ calculated according to ref 35. In addition, by comparing to the formation energies of two holes, we find a binding energy of 0.37 eV per charge for the dimer, again including the finite-size energy correction. The binding energies of STE1 (0.04 eV) and STE2 (0.01 eV) are reported in ref 23. A comparison of these results suggests a much stronger binding of the O–O hole dimer than STE1 or STE2.

**Figure 3:**
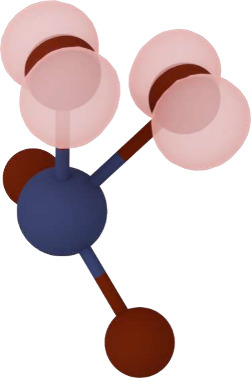
Drawing of the dimer on a VO_4_ unit found in BiVO_4_ with its charge densities. V and O atoms are colored in dark blue and dark red, respectively, while the charge isosurfaces for the holes are shown in pink.

The activation barrier for dimer formation (option iv) results in the highest calculated value up to now (see Figure S4a). With 389 meV, the energy required for it is larger than any of the STE processes (Figure 2c). The dissociation of this arrangement into two separated holes requires an energy of 1071 meV, almost 1 eV higher than any other investigated process. Trapping an electron at the dimer (option v) is accomplished with a very low barrier of 42 meV (see Figure S4b), while releasing (detrapping) the same electron from the dimer configuration is found at 377 meV, still higher than any STE process (Figure 2c). All of the calculated energies are compared in Table 1. The energy barrier for dimer formation (option iv) leads to a microsecond time delay, which is within the range of 10 ns to 10 μs for the trap-limited recombination reported by Ravensbergen et al.^16^ The calculated delay for dissociation is extremely long with several hours, as expected from the high barrier, and confirms the high stability of the dimer. Once the described dimer has formed, it can trap an electron within subpicosecond time scale, similar to STE1 formation. Releasing (detrapping) that additional electron amounts to a time delay on the nanosecond scale. Again, this is within the range of the time delay for trap-limited recombination of ref 16.

To conclude, in this work we quantified activation barriers for the mobility, dissociation and transformation of two distinct self-trapped excitons (STEs) in BiVO_4_, enabling estimates of the associated kinetic time scales.

Our analysis reveals significant differences in the stability and mobility of these STEs. The more compact STE2 is more stable and must undergo a multistep dissociation process (with a transition to STE1 first), while the more separated STE1 can dissociate in a single step. The hopping barrier of STE1 is notably lower than that of STE2, reflecting their distinct migration mechanisms: hopping of STE1 involves a temporary delocalization of the hole followed by electron migration, whereas STE2 requires a more complex and energetically demanding separation and retrapping of the electron–hole pair. However, for both STE1 and STE2, dissociation is energetically favored over whole-STE hopping, as the dissociation barriers are lower than the corresponding migration barriers. As a result, both STE types are expected to predominantly dissociate into separated polarons rather than contribute significantly to long-range charge transport via hopping. This is consistent with the general picture that carrier separation and independent polaron transport dominate in biased photoanodes.^26,36^ While dissociation dominates under the conditions considered here, whole-STE hopping may become relevant in systems where the STE binding energy is sufficiently strong or the migration barrier is lower than the dissociation barrier, allowing hopping to outcompete dissociation and recombination. Such conditions may arise in regions of low electric field (e.g., outside the space-charge layer) or in materials with suppressed free-carrier generation due to strong electron–phonon coupling.^37,38^

The transformation between the two STE types further highlights their distinct behaviors. The transition from STE2 to STE1 requires a higher activation energy, reflecting the need to separate the electron–hole pair before hole relocalization. Conversely, the reverse process—from STE1 to STE2—has a lower barrier, as it primarily involves the recombination of the hole with the electron. We note that the difference between the barriers exceeds the difference in formation energies, indicating that the latter is not a sufficient estimate for STE mobility.

Additionally, our investigation of the formation of the O–O hole dimer and its interaction with single electron polarons offers an alternative mechanism for charge trapping. The high activation barriers for dimer formation and electron release suggest that these processes occur on much longer time scales than STE dynamics, providing an alternative trapping pathway with substantially slower kinetics that may contribute to long-lived trapped charge populations observed experimentally.^16^

Overall, our results provide a comprehensive understanding of the mobility, dissociation and transformation pathways of STEs and O–O dimers in BiVO_4_. These insights are crucial for elucidating the fundamental limitations of charge transport in this material and for guiding the development of strategies to enhance its photoelectrocatalytic performance.

## Supplementary Material



## Data Availability

Structures and input files needed to reproduce the results are available on Zenodo at 10.5281/zenodo.18546285.

## Notes

Detailed computational parameters, descriptions of the computational methods, convergence tests between CP2K and different VASP settings, detailed explanations of the investigated processes and additional figures showing the NEB pathways of the barriers and isosurfaces (PDF)

## References

[1] Bolton J. R., Strickler S. J., Connolly J. S. (1985). Limiting and realizable efficiencies
of solar photolysis of water. Nature.

[2] Yan Q., Yu J., Suram S. K., Zhou L., Shinde A., Newhouse P. F., Chen W., Li G., Persson K. A., Gregoire J. M., Neaton J. B. (2017). Solar fuels photoanode
materials discovery by integrating
high-throughput theory and experiment. Proc.
Natl. Acad. Sci. U.S.A..

[3] Kamble G. S., Natarajan T. S., Patil S. S., Thomas M., Chougale R. K., Sanadi P. D., Siddharth U. S., Ling Y.-C. (2023). BiVO4 As a Sustainable
and Emerging Photocatalyst: Synthesis Methodologies, Engineering Properties,
and Its Volatile Organic Compounds Degradation Efficiency. Nanomaterials.

[4] Ho-Kimura S. (2024). Experimental
Evidence for Photoactivated BiVO4 Anodes with Enhanced Photoelectrochemical
Water Oxidation. ACS Applied Energy Materials.

[5] Abdi F. F., van de Krol R. (2012). Nature and
Light Dependence of Bulk Recombination in
Co-Pi-Catalyzed BiVO4 Photoanodes. J. Phys.
Chem. C.

[6] Wang S., Wan K., Feng J., Yang Y., Wang S. (2025). BiVO4 photoanodes with
enhanced photoelectrochemical performance: Preparation, modification
and emerging applications. Journal of Materials
Science & Technology.

[7] Kwon J., Choi H., Choi S., Sun J., Han H., Paik U., Choi J., Song T. (2025). Improved Charge Carrier
Dynamics by Unconventional Doping Strategy for BiVO4 Photoanode. Small Science.

[8] Kweon K. E., Hwang G. S., Kim J., Kim S., Kim S. (2015). Electron small
polarons and their transport in bismuth vanadate: a first principles
study. Phys. Chem. Chem. Phys..

[9] Suzuki Y., Murthy D. H. K., Matsuzaki H., Furube A., Wang Q., Hisatomi T., Domen K., Seki K. (2017). Rational Interpretation
of Correlated Kinetics of Mobile and Trapped Charge Carriers: Analysis
of Ultrafast Carrier Dynamics in BiVO4. J. Phys.
Chem. C.

[10] Nakatsukasa Y., Katayama K. (2025). Visualization of Synthesis-Dependent Trapped Charge
Carrier Behavior in BiVO4 and Its Relation to the Performance. J. Phys. Chem. C.

[11] Shluger A. L., Stoneham A. M. (1993). Small polarons in real crystals:
concepts and problems. J. Phys.: Condens. Matter.

[12] Gordeev, G. ; Hill, C. ; Gudima, A. ; Reich, S. ; Guennou, M. Resonant Raman signatures of exciton polarons in a transition metal oxide: BiVO_4_ . 2024; https://arxiv.org/abs/2404.04112.

[13] Jiang X., Cheng X., Liu Z., Ding L., Han W. (2025). First-Principles
Study of Polarons in Multiple Crystal Phases of Bismuth Vanadate. J. Phys. Chem. C.

[14] Ziwritsch M., Müller S., Hempel H., Unold T., Abdi F. F., van de Krol R., Friedrich D., Eichberger R. (2016). Direct Time-Resolved
Observation of Carrier Trapping and Polaron Conductivity in BiVO4. ACS Energy Letters.

[15] Zhang J., Shi J., Chen Y., Zhang K. H. L., Yang Y. (2022). Bimolecular Self-Trapped
Exciton Formation in Bismuth Vanadate. J. Phys.
Chem. Lett..

[16] Ravensbergen J., Abdi F. F., van Santen J. H., Frese R. N., Dam B., van de Krol R., Kennis J. T. M. (2014). Unraveling the Carrier Dynamics of
BiVO4: A Femtosecond to Microsecond Transient Absorption Study. J. Phys. Chem. C.

[17] Wiktor J., Ambrosio F., Pasquarello A. (2018). Role of Polarons in Water Splitting:
The Case of BiVO4. ACS Energy Letters.

[18] Butler K. T., Dringoli B. J., Zhou L., Rao P. M., Walsh A., Titova L. V. (2016). Ultrafast carrier
dynamics in BiVO4 thin film photoanode
material: interplay between free carriers, trapped carriers and low-frequency
lattice vibrations. J. Mater. Chem. A.

[19] Kahraman A., Barzgar Vishlaghi M., Baylam I., Sennaroglu A., Kaya S. (2019). Roles of Charge Carriers
in the Excited State Dynamics of BiVO4 Photoanodes. J. Phys. Chem. C.

[20] Park H. S., Kweon K. E., Ye H., Paek E., Hwang G. S., Bard A. J. (2011). Factors in the Metal Doping of BiVO4
for Improved Photoelectrocatalytic
Activity as Studied by Scanning Electrochemical Microscopy and First-Principles
Density-Functional Calculation. J. Phys. Chem.
C.

[21] Fernandez E. N., van de
Krol R., Abdi F. F. (2024). Tuning the Optical and Photoelectrochemical
Properties of Epitaxial BiVO4 by Lattice Strain. Small Structures.

[22] Li Q., Wang L., Zhang J., Dittrich T., Ni C., Yang Y., Zhao J., Cui J., Li C., Fan F. (2025). Operando Imaging
of Polaron-Mediated Charge Transfer across the Electric
Double Layer of BiVO4. J. Am. Chem. Soc..

[23] Möslinger T., Österbacka N., Wiktor J. (2025). Competing Self-Trapped Exciton States
and Multiple Emission Pathways in BiVO4. J.
Phys. Chem. Lett..

[24] Ambrosio F., Wiktor J. (2019). Strong Hole Trapping Due to Oxygen
Dimers in BiVO4:
Effect on the Water Oxidation Reaction. J. Phys.
Chem. Lett..

[25] Fujishima A., Honda K. (1972). Electrochemical Photolysis of Water at a Semiconductor Electrode. Nature.

[26] Walter M. G., Warren E. L., McKone J. R., Boettcher S. W., Mi Q., Santori E. A., Lewis N. S. (2010). Solar Water Splitting Cells. Chem. Rev..

[27] Kühne T. D. (2020). CP2K: An electronic structure and molecular dynamics software package
- Quickstep: Efficient and accurate electronic structure calculations. J. Chem. Phys..

[28] Henkelman G., Uberuaga B. P., Jónsson H. (2000). A climbing image nudged elastic band
method for finding saddle points and minimum energy paths. J. Chem. Phys..

[29] Palermo G., Falletta S., Pasquarello A. (2024). Migration of hole polarons in anatase
and rutile TiO_2_ through piecewise-linear functionals. Phys. Rev. B.

[30] Marcus R. A. (1993). Electron
transfer reactions in chemistry. Theory and experiment. Rev. Mod. Phys..

[31] Emin D., Holstein T. (1969). Studies of small-polaron motion IV.
Adiabatic theory
of the Hall effect. Annals of Physics.

[32] Holstein T. (1959). Studies of
polaron motion: Part I. The molecular-crystal model. Annals of Physics.

[33] Emin, D. Polarons; Cambridge University Press: 2012.

[34] Franchini C., Reticcioli M., Setvín M., Diebold U. (2021). Polarons in materials. Nature
Reviews Materials.

[35] Falletta S., Wiktor J., Pasquarello A. (2020). Finite-size
corrections of defect
energy levels involving ionic polarization. Phys. Rev. B.

[36] Rettie A. J. E., Lee H. C., Marshall L. G., Lin J.-F., Capan C., Lindemuth J., McCloy J. S., Zhou J., Bard A. J., Mullins C. B. (2013). Combined Charge Carrier Transport and Photoelectrochemical
Characterization of BiVO4 Single Crystals: Intrinsic Behavior of a
Complex Metal Oxide. J. Am. Chem. Soc..

[37] Toyozawa Y. (1958). Theory of
Line-Shapes of the Exciton Absorption Bands. Prog. Theor. Phys..

[38] Stoneham, A. M. Theory of Defects in Solids: Electronic Structure of Defects in Insulators and Semiconductors; Oxford University Press, 2001.

