# Supplementary material for: Evolution of Excited States in Bismuth Vanadate: Trapping and Kinetic Pathways
Source: J Phys Chem Lett. 2026 Apr 10;17(20):5646–51. doi: 10.1021/acs.jpclett.6c00396 (PMC13200253; doi:10.1021/acs.jpclett.6c00396)
Supplement: Supplementary file 1 [file jz6c00396_si_001.pdf]

Supporting Information: Evolution of Excited States in Bismuth Vanadate: Trapping and Kinetic Pathways

Tobias Möslinger* and Julia Wiktor*

Department of Physics, Chalmers University of Technology, SE-412 96 Gothenburg, Sweden

E-mail: tobias.moeslinger@chalmers.se; julia.wiktor@chalmers.se

Computational Details

Throughout this project, DFT calculations were performed using the CP2K code and VASP. While the former was used to analyze the formation of STEs in the material and to calculate all the shown paths for modifications of the configurations in NEB computations, the latter was applied to verify the results and guarantee the desired accuracy.

CP2K

The $BiVO_4$ unit cell consists of 24 atoms with a lattice vector of $a = 5.2$ Å, $b = 5.2$ Å, and $c = 11.8$ Å. For all calculations, a $2\sqrt{2}\times2\sqrt{2}\times1$ supercell containing 192 atoms was used, for which the formation energies of STEs are shown to be converged within 0.1 eV in Ref. S1. We used GTH-PBE pseudopotentials and DZVP-MOLOPT-SR-GTH basis sets for all elements. A Gaussian and plane waves (GPW) approach to density functional theory was implemented. The valence states were expanded in a double-zeta valence polarized MOLOPT basis set and an auxiliary plane-wave basis with a cutoff energy of 800 Ry was applied. To describe the exchange-correlation effects, the PBE0-TC-LRC hybrid functional with 14 % exact exchange and a truncated Coulomb potential with a cutoff of 5 Å was used. The α parameter was determined from Koopmans' condition for the electron polaron in Ref. S1. We used the auxiliary density matrix method together with element-specific auxiliary basis sets in order to accelerate the hybrid functional calculations. Spin polarization was considered through the local spin density (LSD) approximation. Fixed-cell geometry optimizations were performed until the energy and force convergence criteria of 5×10^{-7} Ha and 0.01 eV/Å $= 2 \times 10^{-4}$ Ha/Bohr, respectively, were satisfied. The sampling of the Brillouin zone was carried out at the Γ-point.

To determine the energy barriers for the migration and dissociation of STEs, we first fully relaxed the initial and final states of interest. Then, we performed NEB calculations with

five intermediate images generated by CP2K. The climbing image (CI)-NEB method[S2] was used to refine the highest-energy image along the reaction path, ensuring an accurate determination of the transition state. The force convergence criterion for the NEB calculations was set to 4.5×10^{-4} Ha/Bohr.

VASP

To verify the dependence of energy barriers on the k-point sampling, we performed additional calculations within the Vienna Ab-initio Simulations Package (VASP).[S3] These calculations consisted of VASP single-point energies for the initial and final NEB structures and for the highest-energy image along the CP2K NEB path, for three selected processes: STE1 hopping, STE1 dissociation, and the transformation from STE2 to STE1. We employed the PBE0(α) functional, with the same value of $\alpha = 14\,\%$ as before. The plane-wave kinetic energy cutoff was set to 300 eV. Projector augmented wave (PAW) potentials were applied to treat 5 valence electrons for Bi and V and 6 for O. Similarly to before, spin polarization was included to account for unpaired electron states like STEs, while the structural geometries were imported from the CP2K calculations without any modifications or further relaxation. The Brillouin zone sampling was performed using Γ-centred k-point meshes for both the Γ-point only as well as a $2 \times 2 \times 2$ grid. A smearing with a width of 0.0001 eV was applied.

Method for Finding STE Configurations

The generation of structures for the two types of STEs in $BiVO_4$ were accomplished by displacing the closest oxygen neighbours. For the electron, located around the V atom, the four closest oxygen atoms were moved about 0.2 Å further away, whereas for the hole located around the Bi atom, the eight closest oxygen atoms were moved around 0.2 Å closer. This resulted in the STE1 type (as presented in Ref. S1). The STE2 type presented there required a more careful displacement of the neighbouring oxygen atoms. As in this case the electron

and hole both localize around the V atom, only the four closest oxygens needed to be moved, namely in a way that increases the distance to the V slightly by about 0.05 Å to 0.1 Å for three of them and 0.3 Å for the fourth while reducing two angles between oxygens next to each other and therefore increasing the other angles.

Convergence of Calculated Energies and Barriers

A comparison between Brillouin zone sampling using only the Γ-point and a $2 \times 2 \times 2$ grid showed small differences of about 10 meV (see Table S1). We note that the residual forces from the VASP single-point energy barriers were found at an average of $\pm 0.15\,\mathrm{eV/Å}$, with a maximum of $\pm 0.5\,\mathrm{eV/Å}$. However, a comparison between the residual forces of the sampling of only the Γ-point and the $2 \times 2 \times 2$ grid showed very small deviations of $\pm 0.1\,\mathrm{eV/Å}$. We therefore concluded that Γ-point-only sampling provides sufficiently converged relative energy barriers and is adequate for predicting NEB barriers in this system. Furthermore, the results show that the energies from the VASP calculations are comparable to those from CP2K, with a deviation of around 10 to 20 meV up to a maximum of around 30 meV. All of these differences are within the limit for the accuracy of such DFT calculations. Therefore, for further calculations and analyses, CP2K with only Γ-point sampling was used.

Table S1: Comparison between energy barriers calculated within CP2K and VASP as well as with a denser *k*-point grid for selected processes.

**Type of process**	E_{CP2K} **[meV]**	$E^{\Gamma}_{\mathrm{VASP}}$ **[meV]**	$E^{2x2x2}_{\mathrm{VASP}}$ **[meV]**
STE1 hopping	123	137	150
STE1 dissociation	48	34	46
STE2 to STE1	174	140	148

For all the described paths, the activation barrier was calculated using the NEB method. Therefore, the initial and final configurations were first fully relaxed and then used as inputs

for the NEB calculations, in which CP2K generated five intermediate images to construct an initial path for the process. In the NEB calculations (also carried out using CP2K), we performed a minimum of ten steps for each path and considered the calculation converged when the change in the total energy of the system between consecutive steps was below 10 meV. The resulting NEB paths are shown in Section Energy Barriers of the Investigated Paths.

To clarify the influence of the choice of exact exchange in the hybrid functional, we also performed two NEB calculations using $\alpha = 22\,\%$ instead of $\alpha = 14\,\%$ as before (see Table S2). Due to the lower formation energies and therefore higher stability, the hopping barrier of STE1 is about 60 meV higher with $\alpha = 22\,\%$. Furthermore, due to the more pronounced ordering of the formation energies, with STE2 about 160 meV lower than STE1,[S1] the barrier for transformation from STE2 to STE1 is now almost twice as high (increase of 150 meV), while the opposite direction (STE1 to STE2) requires around 85 meV less energy and therefore is even more likely. However, the qualitative trend obtained using $\alpha = 22\,\%$ remains the same as with $\alpha = 14\,\%$.

Table S2: Comparison between energy barriers calculated with $\alpha = 14\,\%$ and $\alpha = 22\,\%$ for selected processes using CP2K.

Type of process	$E_{\alpha=14\,\%}$ [meV]	$E_{\alpha=22\,\%}$ [meV]
STE1 hopping	123	182
STE2 to STE1	174	324
STE1 to STE2	113	28

Description of the Investigated Paths

As listed in the main text, in total six activation barriers were calculated by performing NEB calculations for the paths of the different processes. These are described here in more detail, and a sketch for each of them is provided.

STE1 hopping The electron and hole are localized in the STE1 state. Both the electron and the hole move from their initial positions to a closest neighbouring V and Bi atom, respectively. The final configuration does not differ from the original one, the STE is still in the same STE1 state. Only the location of the STE has changed. This process is the shortest possible translation that can be achieved without changing the configuration of the STE. The sketch is given in Fig. S1a.

STE2 hopping The electron and hole are localized in the STE2 state. Both carriers migrate together from their initial localization around a V atom to the nearest-neighbour V site, while retaining the STE2 configuration. This is the STE2 analogue of the process described above and corresponds to the shortest translation of the intact STE. The sketch is shown in Fig. S1b.

STE1 dissociation The electron and hole are first localized in the STE1 state. The electron stays in the same position and configuration on a V atom throughout the process, while the hole moves from the initial location around the closest neighbouring Bi atom to the second-closest Bi neighbour. The final and initial Bi atoms are chosen to be first neighbours, which means that the smallest possible translation of the hole is accomplished. The whole process therefore resembles the smallest possible change to separate electron and hole from each other. The sketch is shown in Fig. S1c.

Transformation STE2 to STE1 The electron and hole are initially localized in the STE2 state. During the transformation, the electron remains on the same V site but changes from the STE2 to the STE1 configuration, while the hole detaches from the V and localizes on the nearest Bi site, yielding the STE1 arrangement. This pathway therefore captures both the STE2-to-STE1 transformation and the minimal separation of the hole from STE2. A sketch is shown in Fig. S1d.

Transformation STE1 to STE2 The electron and hole are localized in the STE1 state. The electron stays in the same position on a V atom, however, it changes its configuration from the STE1 shape into the STE2 shape. At the same time, the hole moves from its initial Bi and localizes on the V with the electron, while also changing its configuration from the STE1 to the STE2 shape. The final arrangement is the STE2. This process shows the transformation between STE1 and STE2 and is the reverse of the previously described transformation from STE2 to STE1. It is sketched in Fig. S1e.

Dimer formation Two holes are located around two second-neighbour Bi atoms. The whole system is therefore in a +2 state. Both holes move towards the VO_4 dimer unit, where they localize on two neighbouring O atoms to form the O–O hole dimer. The sketch for this process is shown in Fig. S1f.

Additional electron on dimer The previously described O–O hole dimer can trap an additional electron around the V atom. While the whole system is now in a +1 state, the dimer is retained through keeping the -1 (electron) + +2 (hole) combination via multiplicity. When de-trapping (releasing) the electron, it moves from the dimer VO_4 unit to the closest neighbouring VO_4 unit, while the holes stay unchanged in the dimer configuration. This yields a separated electron polaron while the O–O hole dimer remains intact. A sketch is presented in Fig. S1g.

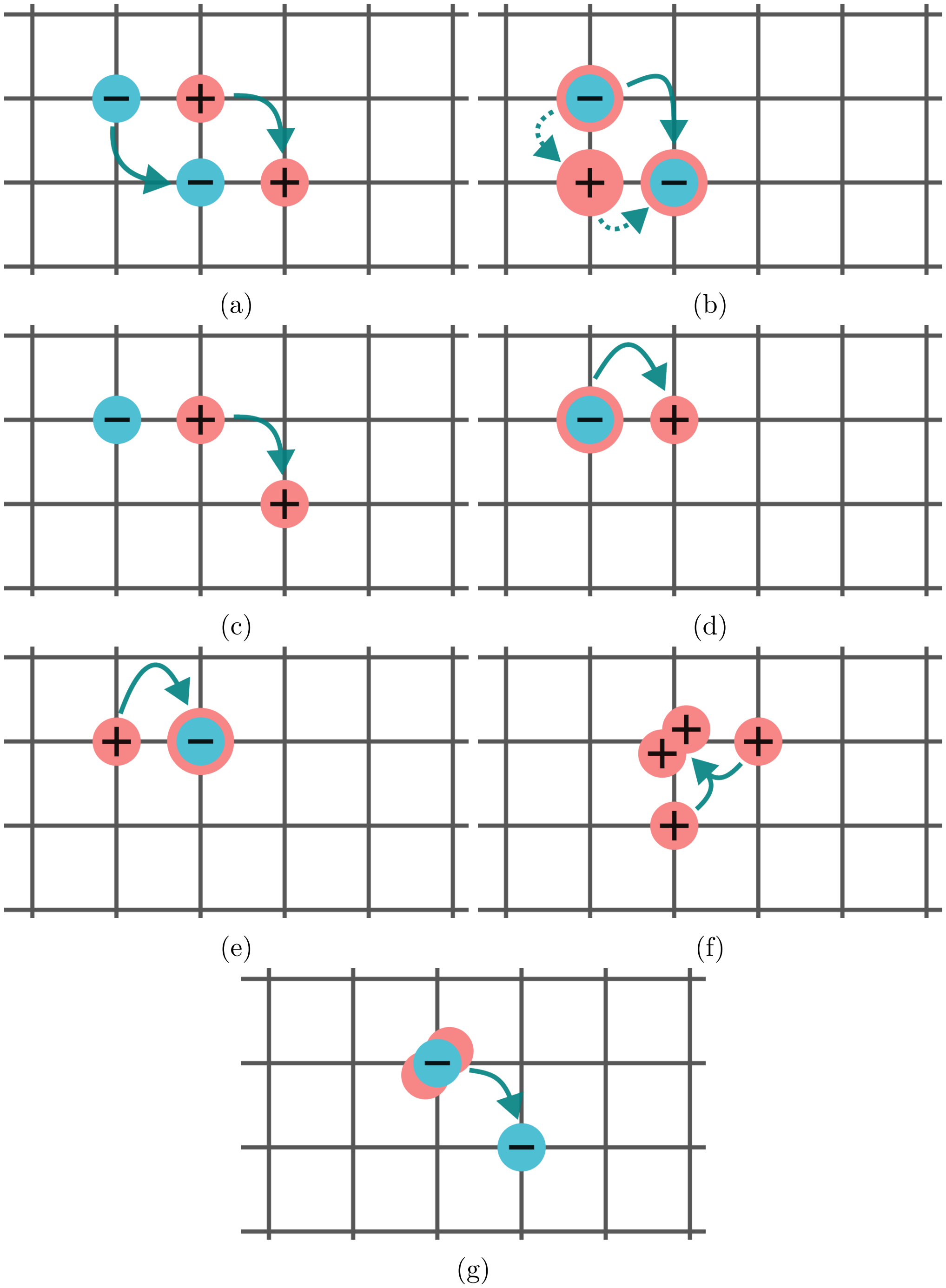

Figure S1: Sketches of the different investigated paths: (a) STE1 hopping, (b) STE2 hopping, (c) STE1 dissociation, (d) STE2 to STE1, (e) STE1 to STE2, (f) Dimer formation, (g) Separation of electron from dimer.

Energy Barriers of the Investigated Paths

The energy barriers analyzed in this study and presented in the main paper are shown in more detail in Figures S2, S3 and S4 below, including insets of the initial and final structure with charge isosurfaces.

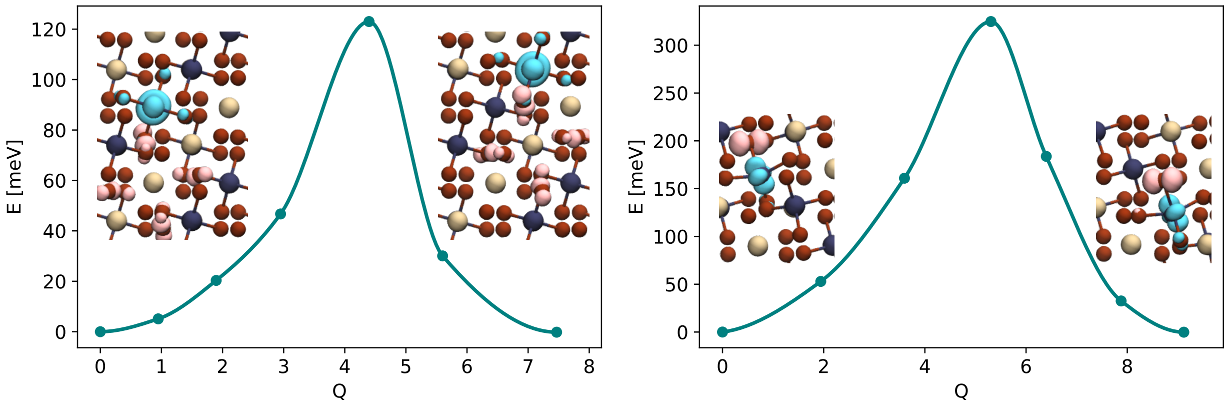

(a) (b)

Figure S2: Plots of the activation barrier for hopping of STE1 (a) and STE2 (b). The insets show the structure and shape of electron (turquoise) and hole (pink) charge isosurfaces in initial and final configuration, with Bi, V and O in beige, dark blue and dark red, respectively.

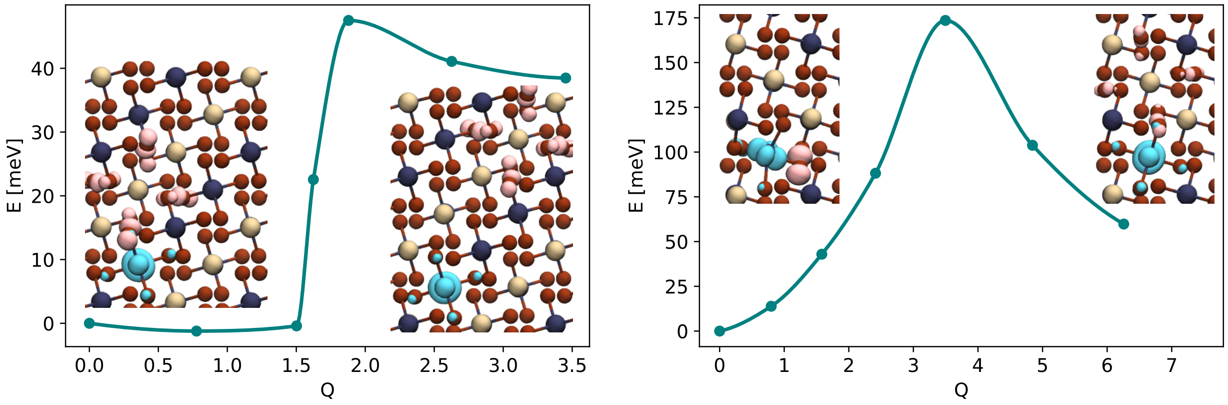

(a) (b)

Figure S3: Plots of the activation barrier for dissociation of STE1 (a) and transformation from STE2 to STE1 (b). The insets show the structure and shape of electron (turquoise) and hole (pink) charge isosurfaces in initial and final configuration, with Bi, V and O in beige, dark blue and dark red, respectively.

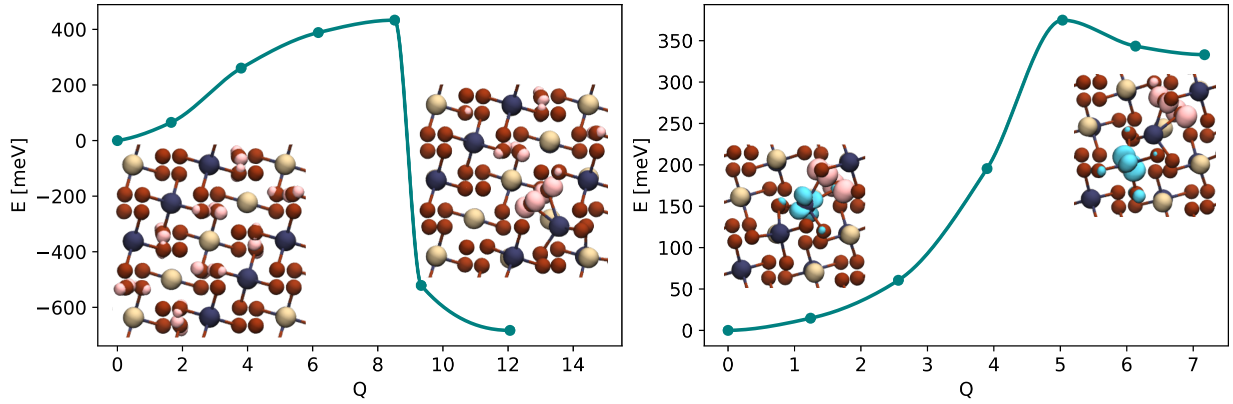

(a) (b)

Figure S4: Plots of the activation barrier for dimer formation (a) and electron break-off (b). The insets show the structure and shape of electron (turquoise) and hole (pink) charge isosurfaces in initial and final configuration, with Bi, V and O in beige, dark blue and dark red, respectively.

Detailed Time Delays of Investigated Processes

The time delays were calculated according to the method described in the main paper. The exact results are presented together with the corresponding energy barrier in Table S3. However, it should be noted that due to the described assumptions taken for the calculation of these values, they only give an approximate estimate of the time delay. Therefore, our discussion is only based on them at an order-of-magnitude level.

Table S3: Energy barriers for different hopping, dissociation or transformation mechanisms and their corresponding time delays. All calculations for are done with CP2K.

Type of process	E [meV]	τ
STE1 hopping	123	15 ps
STE2 hopping	322	30 ns
STE1 formation	9	0.1 ps
STE1 dissociation	48	0.5 ps
STE2 to STE1	174	70 ps
STE1 to STE2	113	5 ps
Dimer formation	389	1230 ns
Dimer separation	1071	250 min
Dimer - electron trapping	42	0.3 ps
Dimer - electron separation	377	230 ns

References

(S1) Möslinger, T.; Österbacka, N.; Wiktor, J. Competing Self-Trapped Exciton States and Multiple Emission Pathways in BiVO4. *The Journal of Physical Chemistry Letters* **2025**, *16*, 6861–6865, PMID: 40569079.

(S2) Henkelman, G.; Uberuaga, B. P.; Jónsson, H. A climbing image nudged elastic band method for finding saddle points and minimum energy paths. *The Journal of Chemical Physics* **2000**, *113*, 9901–9904.

(S3) Hafner, J.; Kresse, G. *Properties of Complex Inorganic Solids*; Springer US: Boston, MA, 1997; pp 69–82.
